# Mutual entailment between causation and responsibility

**DOI:** 10.1007/s11098-023-02041-2

**Published:** 2023-11-20

**Authors:** Justin Sytsma, Pascale Willemsen, Kevin Reuter

**Affiliations:** 1 Philosophy Programme, Victoria University of Wellington, Wellington, 5036 Aotearoa New Zealand; 2 Department of Philosophy, University of Zurich, 8044 Zurich, Switzerland

**Keywords:** Entailment claim, Causation, Responsibility, Experimental philosophy, Causal cognition

## Abstract

The standard view in philosophy is that responsibility entails causation. Most philosophers treat this entailment claim as an evident insight into the ordinary concepts of responsibility and causation. Further, it is taken to be equally obvious that the reversal of this claim does not hold: causation does not entail responsibility. In contrast, Sytsma and Livengood have put forward an account of the use of ordinary causal attributions (statements like “X caused Y”) that contends that they are typically used interchangeably with responsibility attributions (statements like “X is responsible for Y”). Put in terms of the concepts at play in these attributions, this account suggests that the reversal of the entailment claim may also hold, and, a fortiori, there would be mutual entailment between the ordinary concepts of responsibility and causation. Using the cancellability test, we report the results of three pre-registered studies providing empirical evidence that causation and responsibility are mutually entailed by each other.

## Introduction

Sartorio (2007, p. 750) writes, “here’s a very natural idea about the relation between moral responsibility for outcomes and causation: moral responsibility for an outcome requires causing it”. This expresses a common claim about the relationship between responsibility and causation in philosophy. Call this the Entailment Claim. This claim holds that causing an outcome is a necessary, conceptual precondition for being responsible for that outcome (see also, Driver, 2007; Sartorio, 2007; Scanlon, 2008; Wolf, 1993). The Entailment Claim seems plausible. Afterall, it seems that we can hardly blame a person for a broken window, if that person did not cause the window to break.^1^ However, causation is standardly thought to be insufficient for responsibility: even if a person caused the window to break, we might not judge that they are responsible for that outcome, e.g., if the person was not in control of what they were doing.

Philosophers have proposed different versions of the Entailment Claim, with some of them including or excluding omissions, making additional grounding claims, and so on.^2^ There are four key claims that unite most of these proposals. First, the concept of causation at issue is not supposed to be a technical one, but to reflect the concept employed in ordinary causal judgments. Some authors commit explicitly to the idea that an adequate theory of causation must capture our ordinary understanding (for discussion see Livengood et al., 2017). For instance, in defending causation by omissions, Schaffer (2004, p. 205) states that “to dismiss negative causation is to swallow” that “the folk are wrong that voluntary human action is causal, the law is wrong that negligence is causal, ordinary language is wrong that ‘remove’, ‘release’, ‘disconnect’, and so on are causal”; based on this, he goes on to “submit that no theory so dismissive deserves to be considered a theory of causation”. The close connection between theories of causation and our ordinary understanding of causation is further illustrated by the plethora of thought experiments employed in the literature to elicit our causal intuitions.^3^

Second, philosophers often assume, whether implicitly or explicitly, that the ordinary concept of causation is expressed most fundamentally by the use of the lemma “cause” in making causal attributions—in issuing verdicts such as “X *caused* Y” or “X is the *cause* of Y” (e.g., Driver, 2007; Sartorio, 2007; Skow, 2019).^4^ Put another way, the concept of causation at issue in these discussions is the one that is typically at play in ordinary causal attributions.

Third, it is generally taken for granted that there is an important conceptual and metaphysical distinction between causation and moral responsibility, such that it makes sense and seems philosophically fruitful to investigate their relationship.

Fourth, and relatedly, it is typically assumed that while normative considerations play a central role in the applicability of the concept of moral responsibility, the concept of causation is independent of such considerations, merely describing the causal chain that led to the outcome.^5^ In other words, the concept of causation is taken to be purely descriptive. Indeed, this is a cornerstone in these debates, as causation is often thought of as explaining, justifying, or grounding responsibility. The possibility of such a grounding relation, however, requires the independence of the grounding and the grounded (see Sartorio, 2007). Thus, whatever norms are at play in (correctly) applying the concept of responsibility, they should play no such role for (correctly) applying the concept of causation.

Taken together, these four claims inform the standard view of the relationship between causation and responsibility that we are concerned with here:

The standard view holds that causation is a necessary, but not sufficient, precondition for moral responsibility, and assumes that the concept of causation at issue corresponds with the ordinary concept, which is the one canonically expressed by attributional uses of the lemma “cause”, is a distinct concept from moral responsibility, and is purely descriptive.

We are skeptical of this standard view. One reason to doubt the standard view comes from recent work in the experimental literature on causal attributions. This research has shown that normative considerations, including moral considerations, have a notable influence on the causal attributions people endorse.^6^ A number of different explanations of these findings have been put forward, typically attempting to square the effect with the assumption that the ordinary concept of causation is purely descriptive.^7^

One leading explanation takes another tact, however. The *responsibility account*, first put forward in Sytsma et al. (2012), contends that normative considerations impact causal attributions because such attributions are not typically used to simply express descriptive judgments, but also to express normative judgments. On this account, in ordinary English, statements like “X caused Y” are standardly used to indicate something more than that someone (or something) brought about an outcome, or contributed to bringing about an outcome: they also express a normative evaluation concerning accountability for that outcome. As such, if we follow the literature in taking the ordinary concept of causation to be the one at play in the dominant use of causal attributions, then the responsibility account holds that the ordinary concept of causation is not purely descriptive, but has indispensable evaluative content.^8^

The responsibility account predicts that the phrases “X caused Y” and “X is responsible for Y” will commonly be used interchangeably. This means that when an individual asserts that “Steve caused the window to break”, according to the responsibility account, they could just as well have stated that “Steve is responsible for the window getting broken”, and vice versa. Not only do both statements express that there is a descriptive connection between the agent Steve and the broken window, but each also conveys a normative assessment. Specifically, they indicate that Steve is accountable for the occurrence of the broken window. Nonetheless, it is important to note that the responsibility account does not exclude the possibility that the use of such statements may differ in terms of the aspect of the relationship that is more strongly emphasized.

The responsibility account pushes against another piece of philosophical orthodoxy, beyond the key claims uniting the standard view noted above. It is commonly assumed that there are multiple ordinary concepts of responsibility. Most importantly, while philosophers often use “responsibility” synonymously with “moral responsibility”, they sometimes distinguish this normative concept from a purely descriptive concept of *causal responsibility*. Sytsma (2022b) raises doubts about whether this distinction reflects the ordinary use of responsibility attributions, suggesting that the dominant use is normative, but not necessarily moral. The idea is that attributing responsibility ordinarily goes beyond the purely descriptive, but that the norms at play might fall short of what one is inclined to label as *moral*. For present purposes, the key point is that while the responsibility account focuses on a comparison to responsibility attributions (statements like “X is responsible for Y”), its proponents understand such attributions in a way that aligns with the concept of moral responsibility at issue for the Entailment Claim. We return to this issue in Sect. 3.

There is now a good deal of evidence supporting the responsibility account, including studies indicating that people’s judgments about causal attributions are quite similar to their judgments about normative claims like responsibility attributions and blame attributions.^9^ This suggests that not only will people tend to treat causation as being necessary for responsibility, but that they’ll tend to treat responsibility as being necessary for causation. Taking the responsibility account to illuminate the ordinary concepts of causation and responsibility, it holds that these concepts are much more similar than the standard view supposes. This, in turn, suggests not only that people will tend to treat responsibility as entailing causation (the Entailment Claim that the standard view supposes) but that they’ll also tend to treat causation as entailing responsibility (the Reverse Entailment Claim the standard view denies). In other words, the responsibility account suggests that the conceptual relationship between responsibility and causation is one of *mutual entailment*.

In the next two sections, we present the results of three studies designed to test the twin entailment claims comprising mutual entailment. In all three studies, we take a rather novel methodological approach. While most of the experimental work on the ordinary concept of causation features vignette-based studies, we instead use the cancellability test in this paper. Although the cancellability test is widely known in the philosophical and linguistic literature, it has only recently been applied to *empirically* investigating semantic-cum-pragmatic relations between concepts in philosophy (Willemsen & Reuter, 2021; Baumgartner et al., 2022; Coninx et al., 2023; Almeida et al., 2023). In the final section we then turn to potential objections, reporting the results of a fourth study testing a worry about our use of the cancellability test.

## Study 1: testing mutual entailment between responsibility and causation

The cancellability test is used to examine whether a feature or component is conversationally implicated by another concept (Grice, 1989). For instance, by saying “I tried to publish a book”, a speaker usually conveys the additional information that they failed to do so. However, canceling this derived piece of information does not result in a contradictory statement: “I tried to publish a book, but by that I am not saying that I failed to do so” sounds perfectly fine since the speaker might simply want to highlight the attempt. However, some pieces of information cannot be canceled in the same way. For instance, saying “Tom is a bachelor, but by that I am not saying that he is unmarried” is contradictory, as being unmarried is semantically entailed by being a bachelor.

In the studies in this paper, we used the cancellability test to investigate mutual entailment, assessing the competing claims of the standard view and the responsibility account. In our first study, we assume the contention from advocates of the responsibility account noted above that the dominant ordinary concept of responsibility is a normative concept, testing cancellation statements involving “caused” and “responsible”.

### Materials and methods

Each participant in our study judged whether each of five statements was contradictory. This set was comprised of two key test statements—one to test the Entailment Claim (EC) and one to test the Reverse Entailment Claim (REC)—and three comparison statements (Control, Semantic Entailment, Conversation Implicature). Each statement was prefaced by telling participants to “please imagine that Sally said the following sentence”. The five statements were presented in random order and were preceded by a short training round explaining the notion of contradiction and giving participants two practice questions. Methodology and hypotheses were pre-registered at the Open Science Framework and full materials can be accessed through the online repository.^10^

The EC, REC, and Control statements each involve a causal attribution concerning one of three agents bringing about a different outcome:

i. John caused the file to be deleted
ii. Brian caused the patient to get worse
iii. Steve caused the window to break

These attributions were assigned randomly, such that each participant received a statement using each of the three without repetition. For the EC statements, responsibility is asserted and causation is denied. To illustrate, for (i) the corresponding EC statement is:

John is responsible for the file being deleted, but by that I am not saying that John caused the file to be deleted.

The REC statements reverse this, with causation being asserted and responsibility denied. To illustrate, for (ii) the corresponding REC statement is:

Brian caused the patient to get worse, but by that I am not saying that Brian is responsible for the patient getting worse.

After each statement participants were asked “Does Sally contradict herself?” and answered using a 9-point scale anchored at 1 with “definitely not” and at 9 with “definitely yes”.

The purpose of the Control statements is to test whether participants treat just any attribution as being entailed by the causal claim. To do this, the Control statements assert causation (like the REC statements), but now deny that the agent *wanted* that outcome to occur. To illustrate, for (iii) the corresponding Control statement is:

Steve caused the window to break, but by that I am not saying that Steve wanted the window to break.

Since an agent causing an outcome does not necessarily mean that the agent wanted that outcome to occur, we predicted that responses to the Control statements should be low.

The other two statements—Semantic Entailment and Conversational Implicature—were used to set a high and low baseline for comparison, respectively. Semantic Entailment reads as follows:

This is a lake, but by that I am not saying that it consists of water.

This statement gives an example where the assertion and denial are contradictory on the dominant use of the terms at issue. As such, we expected contradiction ratings to be high. By contrast, Conversational Implicature gives an example where what is denied is implied by the person making the assertion, but is not entailed by the statement:

This chocolate is good value-for-money, but by that I am not saying that we should buy it.

Here we expected that contradiction ratings should be low.

### Hypotheses and participants

There are three key null hypotheses for the EC and REC test statements that are relevant to testing the standard view and the responsibility account:

#### Hypothesis 1

Average contradiction ratings for EC statements are not significantly above the midpoint.

#### Hypothesis 2

Average contradiction ratings for REC statements are not significantly above the midpoint*.*

#### Hypothesis 3

No significant difference in contradiction ratings for EC statements and REC statements.

As discussed above, both the standard view and the responsibility account hold that responsibility entails causation on the dominant ordinary concepts. This means that both views predict that people will tend to judge that the EC statements are contradictory, since responsibility is asserted while causation is denied. As such, both views expect Hypothesis 1 to be rejected. By contrast, the standard view and the responsibility account diverge with regard to whether causation entails responsibility: the responsibility account holds that it does, while the standard view denies this. This means that while the responsibility account predicts that people will tend to judge that the REC statements are contradictory, the standard view predicts that people will tend to judge that they are non-contradictory. As such, the responsibility account predicts that Hypothesis 2 will be rejected while the standard view predicts that it will not be rejected.

It follows from these first two predictions that advocates of the standard view expect rather different responses for EC and REC statements, while advocates of the responsibility account expect these to be similar. As such, the standard view predicts that Hypothesis 3 will be rejected while the responsibility account predicts that it will not be rejected. Although Hypothesis 3 is not an imperative for mutual entailment, it is indeed an outcome that arises naturally from the responsibility account. According to the responsibility account, the expressions “X caused Y” and “X is responsible for Y” are typically used interchangeably. From this it would follow that insofar as participants read the attributions in our cancelation statements in their dominant senses, the contradiction ratings should be similar. Thus, even though Hypothesis 3 presents a stronger prediction for the responsibility account than the other two hypotheses, it is one that follows from a robust version of this account.

Participants were recruited through Prolific and reimbursed for their participation (pre-selection criteria: Approval Rate > 90%, Native Language English, Age 18). Results were collected from 71 participants (62.0% women, one non-binary, average age 31.0 years).

### Results

**Fig. 1:**
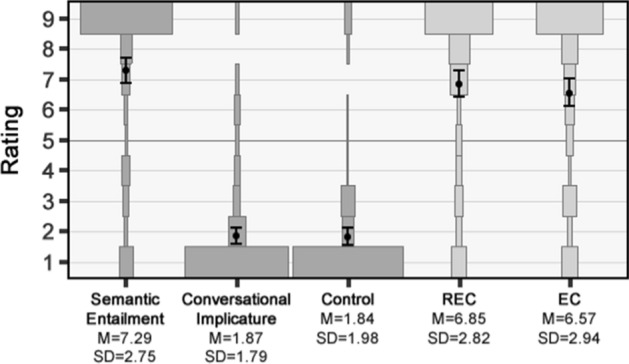
Results of study 1. Plots show the relative percentage of participants selecting each response option, with means (dots) and 95% confidence intervals overlaid (error bars)

The mean contradiction ratings for the five questions in the main study are depicted in Fig. 1 along with the distribution of responses. Responses for the two practice questions indicated that participants understood the idea of a speaker contradicting herself.^11^ The baseline and control conditions worked as expected, with Semantic Entailment having a mean contradiction rating significantly above the mid-point of 5 [*M* = 7.29, *SD* = 2.75; *t*(70) = 7.33, *p* < 0.001, *d* = 0.87], while Conversational Implicature and Control had mean ratings significantly below the mid-point [*M* = 1.87, *SD* = 1.79; *t*(70) = -11.80, *p* < 0.001, *d* = 1.40; and *M* = 1.84, *SD* = 1.98; *t*(70) = -15.32, *p* < 0.001, *d* = 1.82].

We collapsed the data for the different outcomes—(i), (ii), and (iii) from above—in the subsequent analysis as there was no significant difference between them.^12^ In line with the predictions of both the standard view and the responsibility account, the EC statements had a mean contradiction rating significantly above the mid-point [*M* = 6.85, *SD* = 2.82; *t*(70) = 3.44, *p* < 0.001 (one-tailed), *d* = 0.41]. Thus, we can reject Hypothesis 1. More importantly, in line with the prediction of the responsibility account, but contrary to the prediction of the standard view, the mean contradiction rating for the REC statements was also significantly above the mid-point [*M* = 6.57, *SD* = 2.94; *t*(70) = 4.67, *p* < 0.001 (one-tailed), *d* = 0.55]. Thus, we can also reject Hypothesis 2. Indeed, ratings for the REC statements were not significantly different from the upper baseline given by Semantic Entailment [*t*(70) = 1.52, *p* = 0.13, *d* = 0.25]. Finally, in line with the prediction of the responsibility account, but contrary to the prediction of the standard view, we found no significant difference between the mean ratings for the EC and REC statements [*t*(70) = 0.86, *p* = 0.40, *d* = 0.13]. Thus, we cannot reject Hypothesis 3.

### Discussion

The responsibility account suggests that on their dominant ordinary use causal attributions and responsibility attributions are largely interchangeable. This generates the predictions that people should tend to treat the EC statements in our study as contradictory (rejecting Hypothesis 1), that they should tend to treat the REC statements as contradictory (rejecting Hypothesis 2), and further that the responses for these types of statements should be largely similar (affirming Hypothesis 3). Taking the dominant ordinary use of these attributions to express the ordinary concepts of causation and responsibility, these findings then suggest that the relationship between the concepts is one of mutual entailment. In contrast, the standard view holds that the relationship is instead just one of unidirectional entailment, with responsibility entailing causation, but not the reverse. As such, the standard view makes competing predictions to the responsibility account for Hypothesis 2 and Hypothesis 3. The results of our first study bore out all three predictions of the responsibility account and, thus, ran counter the second and third predictions of the standard view.

As noted above, however, the conclusion that these results support mutual entailment assumes that people will tend to understand the responsibility attributions in our EC and REC statements as being normative. And while there is reason to expect this to be the case (Sytsma et al., 2019; Sytsma, 2022b), the claim is controversial. As we’ve seen, advocates of the standard view typically distinguish between a normative concept of responsibility (*moral responsibility*) and a descriptive concept (*causal responsibility*). This distinction sets up a ready response to our first study: if participants did not interpret the responsibility attributions in terms of a relevant normative concept, then our results would not in fact support mutual entailment. Our next two studies address this concern.

## Studies 2a & 2b: testing mutual entailment between blame/fault and causation

It might be objected that our first study suffers from a major flaw, namely that we leave the responsibility attributions underspecified. The result, so the objection goes, is that the REC statements from our first study might have triggered readings of “responsible” that are irrelevant to the discussion at hand.

The first issue is that normative responsibility can be forward- and backward-looking. In a backward-looking sense, an agent might be responsible for something she did. In this case, we are referring to responsibility in the sense of blame and praiseworthiness. However, she might also be normatively responsible in a forward-looking sense that is not concerned with blame and praise but with future duties, such as when we say, e.g., “it is my responsibility as faculty member to attend the departmental meetings”. Note, however, that duties are usually not specified for outcomes, and especially not for negative outcomes like making a patient get worse, breaking a window, or deleting a file. Instead, duties are usually connected to *activities* that bring about *desired* outcomes. As such, it seems quite unlikely that participants interpreted the responsibility attributions in our REC statements in terms of duties.

But even if we can be certain that participants took a backward-looking approach to responsibility, a second issue arises due to the potential ambiguity above: defenders of the standard view often distinguish between *causal responsibility* and *moral responsibility*. A person is considered causally responsible if “she is the (or a) salient cause of—some occurrence or outcome” (Talbert, 2019). In other words, causal responsibility is a close cousin, if not even synonymous (at least on some accounts) with the notion of causation (see Willemsen, 2018 for discussion). If the participants in our first study interpreted responsibility as *causal* responsibility, then the results indeed would not be surprising and would not suggest against the standard view or in favor of the responsibility account.^13^

A final issue is that a third sense of “responsible” is sometimes distinguished from the concepts of causal responsibility and moral responsibility—that of *legal responsibility* (Moore, 2010). Thus, a critic might contend that in our previous study participants might well have interpreted the responsibility attributions in the REC statements as saying that the agents are liable for compensating someone for the broken window, the deleted file, or the patient’s condition. It should be noted, here, that while there is clearly a distinction to be drawn between legal responsibility and moral responsibility, both would seem to be normative concepts that involve further considerations beyond a purely descriptive notion of causation. As such, even if participants read the responsibility attributions in our first study in terms of legal responsibility, our results would arguably still suggest against the standard view and in favor of the responsibility account.

How can we address these concerns? A first thing to note is that while *philosophers* might treat “responsible” simpliciter as being ambiguous between “causal responsibility”, “legal responsibility”, and “moral responsibility”, it is unclear that lay people find it similarly ambiguous. And there is some reason to suspect that they do not. Thus, Sytsma et al. (2019) present corpus evidence suggesting that ordinary responsibility attributions are typically normative, while Sytsma (2022b) presents corpus evidence indicating that “morally responsible” is very rarely used. Expanding on this, we collected data from the Corpus of Contemporary American English (COCA). Table 1 lists the number of hits for a range of phrases plausibly expressing different forms of responsibility. As can be seen from the table, ordinary people do not speak of causal responsibility: Of the mere 32 hits for “causal responsibility” and 4 for “causally responsible”, all but 5 come from academic texts. Uses of “morally responsible” and “legally responsible” are more frequent, although they remain rather uncommon, with only 0.2% of all uses of “responsible” being modified with the adverb “morally”.

**Table 1:** List of the absolute hits and relative percentage for various responsibility phrases on COCA

Moral, legal, and causal responsibility		
Term	Absolute number of hits	Percentage (%)
Responsibility	64109	100.00
Moral responsibility	633	0.99
Legal responsibility	205	0.32
Causal responsibility	32	0.05
Responsible	67355	100.0
Morally responsible	134	0.20
Legally responsible	154	0.23
Causally responsible	4	<0.01

What we find is that non-academics seldom, if ever, use the phrases that philosophers employ to distinguish between concepts of responsibility. This does not necessarily mean that ordinary people lack such concepts, however; it simply means that they don’t express them in this way. Nonetheless, these findings are congruent with the contention that scholars have stipulated new technical notions that go beyond the ordinary sense of “responsible”. Coupled with previous results showing that normative considerations matter for people’s responsibility attributions, this suggests against the worries we’ve raised concerning our first study.

The most direct way of addressing these concerns would be to replace “responsible” with “morally responsible” in the statements from our first study, bringing them in line with standard statements of the Entailment Claim. Given the rather infrequent use of this phrase, however, there is a serious risk that participants would read too much into the use of “morally” if it were included in our test sentences. Luckily, there are alternative terms that can be used to resolve the potential ambiguity of “responsibility” and yet remain congruent with the standard view. This was done in a pair of studies: In Study 2a we replaced “responsible” with “to blame” and in Study 2b we replaced “responsible” with “at fault”.

### Studies 2a: entailment between blame and causation

#### Materials, hypotheses, and participants

The design of Study 2a strictly followed the design of Study 1. The only difference was that the term “responsible” was replaced with “to blame” in the EC and REC statements. For instance, the revised statements for causal attribution (i) now read as follows, with bolding added here to highlight differences and not included in the study:

EC: John is **to blame** for the file being deleted, but by that I am not saying that John caused the file to be deleted.

REC: John caused the file to be deleted, but by that I am not saying that John is **to blame** for the file being deleted.

Again, participants were presented with the question “Does Sally contradict herself” and were instructed to provide their responses on a 9-point Likert scale anchored at 1 with “definitely not” and at 9 with “definitely yes”. The same null hypotheses that were posited in Study 1, were investigated in Study 2a: that the average contradiction ratings for EC statements are not significantly above the midpoint (Hypothesis 1), that the average contradiction ratings for REC statements are not significantly above the midpoint (Hypothesis 2), and that there is no significant difference in contradiction ratings between the EC and REC statements (Hypothesis 3).

The same recruitment method and pre-selection criteria were used as in Study 1. Results were collected from 72 participants (50 women, two non-binary persons, average age 31*.*8 years). As before, the study was pre-registered on OSF.^14^

#### Results

**Fig. 2:**
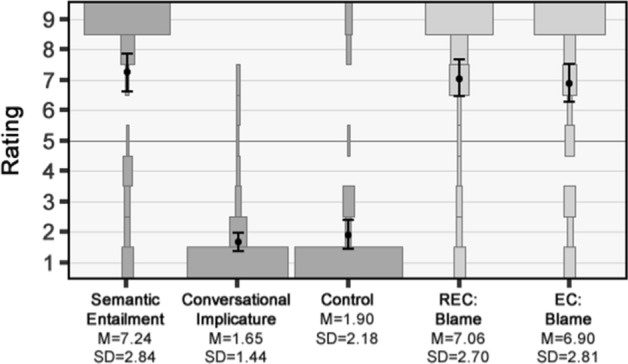
Results of study 2a. The plots show the relative percentage of participants selecting each response option, with means and 95% confidence intervals overlaid

Once again, responses for the two practice questions indicated that participants understood the idea of a speaker contradicting herself.^15^ The mean contradiction ratings for the five questions in the main study are depicted in Fig. 2 along with the distribution of responses. The baseline and control conditions again worked as expected, with Semantic Entailment having a mean contradiction rating significantly above the midpoint [*M* = 7.24, *SD* = 2.84; *t*(71) = 6.69, *p* < 0.001 (one-tailed), *d* = 0.79], while Conversational Implicature and Control had mean ratings significantly below the midpoint [*M* = 1.65, *SD* = 1.44; *t*(71) = −19.78, *p* < 0.001 (one-tailed), *d* = 2.33; *M* = 1.90, *SD* = 2.18; *t*(71) = −12.04, *p* < 0.001 (one-tailed), *d* = 1.42].^16^

The mean contradiction rating for the EC statements was again above the midpoint [*M* = 6.90, *SD* = 2.81, *t*(71) = −12.04, *p* < 0.001 (one-tailed), *d* = 1.42], as was the mean rating for the REC statements [*M* = 7.06, *SD* = 2.70, *t*(71) = 6.47, *p* < 0.001 (one-tailed), *d* = 0.76]. Indeed, once again the mean rating for the REC statements was not significantly different from the upper baseline given by Semantic Entailment [*t*(71) = 0.45, *p* = 0.65, *d* = 0.065]. Finally, as in Study 1, no significant difference was found between the mean ratings for the REC and EC statements [*t*(71) = 0.42, *p* = 0.67, *d* = 0.055].

### Study 2b: entailment between fault and causation

#### Materials, hypotheses, and participants

Study 2b again followed the same structure as Study 1, but this time we replaced “responsible” with “at fault”. For instance, the revised statements for causal attribution (i) now read:

EC: John is **at fault** for the file being deleted, but by that I am not saying that John caused the file to be deleted.

REC: John caused the file to be deleted, but by that I am not saying that John is **at fault** for the file being deleted.

Once again, the same recruitment method and pre-selection criteria were used. Results were collected from 71 participants (64.8% women, two non-binary, average age 28*.*8 years). And, as before, the study was pre-registered on OSF.^17^

#### Results

**Fig. 3:**
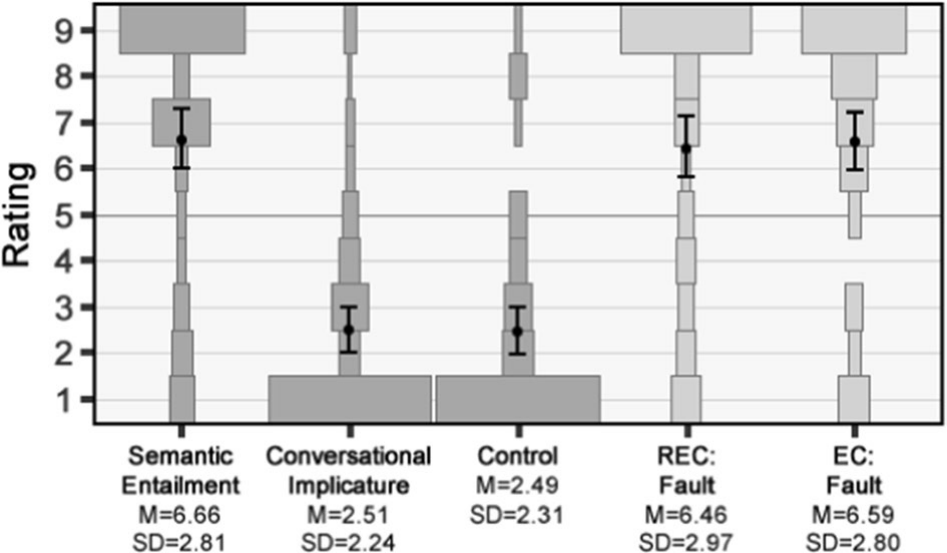
Results of Study 2b. Plots show the relative percentage of participants selecting each response option, with means and 95% confidence intervals overlaid

Results are shown in Fig. 3 and were once again in line with those from Study 1, including for the practice questions, Semantic Entailment, Conversational Implicature, and Control.^18^ As there were no significant effects of the different outcomes for either REC [*F*(2,68) = 0.58, *p* = 0.56, η^2^ = 0.02] or EC [*F*(2,68) = 0.088, *p* = 0.92, η^2^ = 0.003], we again collapsed the data in the subsequent analysis. As in the previous two studies, the mean contradiction rating for the EC statements was above the midpoint [*M* = 6.46, *SD* = 2.97; *t*(70) = 4.79, *p* < 0.001 (one-tailed), *d* = 0.57], as was the mean rating for the REC statements [*M* = 6.59, *SD* = 2.80; *t*(70) = 4.16, *p* < 0.001 (one-tailed), *d* = 0.49]. Indeed, once again the mean rating for the REC statements was not significantly different from the upper baseline given by Semantic Entailment [*t*(70) = 0.45, *p* = 0.66, *d* = 0.068]. Finally, as in the previous studies, no significant difference was found between the mean ratings for the REC and EC statements [*t*(70) = 0.36, *p* = 0.72, *d* = 0.044].

### Discussion

The results of Study 1 suggest that mutual entailment holds between responsibility and causation, providing evidence against the standard view and in line with the responsibility account. Against this interpretation, one might raise the following worry: The term “responsible” is notoriously ambiguous between a duty reading, a descriptive notion, and two backward-looking normative readings. We therefore decided to run two further studies in which we replaced “responsible” with terms that do not readily lend themselves to a non-normative interpretation—“blame” and “fault”. Despite these changes, the results matched those of our first study, and we rejected Hypothesis 1 and Hypothesis 2, but not Hypothesis 3: In line with both views, participants tended to treat each version of the EC statements as contradictory; but, in line with the responsibility account and against the standard view, they also tended to treat the REC statements as contradictory and ratings for these statements were not significantly different from those for the EC statements. Together, the findings across these three studies provide strong evidence that the relationship between the ordinary concepts of responsibility and causation is one of mutual entailment.

## Objections and replies

The standard view claims that responsibility entails causation but that the reverse does not hold. In this paper, we have challenged this view empirically by investigating how the dominant ordinary concepts of causation and responsibility are related. In line with the responsibility account, we hypothesized that not only does causation entail responsibility, but that responsibility also entails causation.

Congruent with both the standard view and the responsibility account, our results suggest that causation is necessary for normative responsibility. Assigning responsibility, blame, or being at fault for one outcome, but immediately denying the agent’s causal involvement is considered highly contradictory. Against the standard view, but in line with the responsibility account, however, participants also considered it to be highly contradictory to attribute causation to an agent, then immediately deny that the agent is responsible, to blame, or at fault for the outcome. This suggests that normative responsibility is also taken to be necessary for causation. As such, these findings jointly indicate that the relationship between responsibility and causation is one of mutual entailment.

To conclude, we consider two related worries that a critic might voice against the evidence we have provided for mutual entailment. Each raises doubts about whether the method we’ve employed—the cancellability test—is able to support our conclusion. As we noted above, the studies presented in this paper diverge from the studies most often employed in looking at the effect of norms on ordinary causal attributions. Such studies present participants with a richer, more elaborate stimulus, most often in the form of a vignette that provides details about a specific scenario. In contrast, the cancellability test doesn’t require such an elaborate stimulus, instead presenting participants with just a simple statement to evaluate. We consider this a strength of the method, since extended vignettes leave more room for misreading and might guide participants toward responses that they might not otherwise give, perhaps by suggesting a non-dominant reading of the key term of interest. Nonetheless, no method is perfect, and while we believe that the cancellability test is an important compliment to other stimulus-based methods, offering the potential for a consilience of evidence, it is not without issues of its own.

### Objection 1: exculpatory context needed

The first worry we’ll discuss contends that what we take to be a strength of the cancellability test is really a weakness. We interpreted high contradiction ratings for our REC statements as evidence that responsibility is not merely conversationally implicated by causation, on the ordinary concepts, but semantically entailed. An anonymous reviewer for *Philosophical Studies*, however, raised the astute point that this inference arguably presumes that if one concept merely conversationally implicates another, contexts in which the former but not the latter applies will tend to come to mind. Against this, though, it can be argued that this might not be the case for causation and responsibility, noting that they will often co-occur, at least in human interactions. And if this is the case for our REC statements, then we would expect our results to obtain even if the Reverse Entailment Claim is false. Further, the critic would expect that if a suitable context were provided to participants, such as one that would morally excuse the agent, we would now expect participants to treat the statements as non-contradictory. Indeed, the reviewer expressed having this intuition for the REC statement and context detailed below.

In response, we have two theoretical reasons for doubting this first objection. First, it isn’t so clear to us that we should expect that potential exculpatory circumstances will not generally come to mind for the types of scenarios we tested. Indeed, we find it unlikely that participants generally wouldn’t come up with potential excuses for *any* of the three statements tested. In each case, it strikes us as rather easy to think of a compelling excuse, from the agent being justifiably ignorant of associated norms, to being tricked, to operating under coercion, and so on. Second, it is not clear that one would need to have a specific exculpatory scenario come to mind to recognize that the statements we tested are non-contradictory if the standard view is correct: if causation is treated as a purely descriptive matter, then it should be clear that it is insufficient for normative responsibility. Recognizing this we would expect people to treat statements asserting the former while denying the latter as non-contradictory even if they are unsure of exactly what details of the situation mitigate the agent’s responsibility for the outcome. Nonetheless, this is an empirical objection, and one that it is worth testing. While a detailed investigation of this worry is beyond the scope of the present paper, we conducted a pilot study to offer a preliminary answer.

Our fourth study used the same basic set-up as our first, but this time we provided brief context for each of the statements. After the training round, each participant was asked to evaluate three statements in random order, one testing Semantic Entailment (SE), one the Entailment Claim (EC), and one the Reverse Entailment Claim (REC). Two options were tested for each type of statement, with each being drawn from our first study (with the exception of a second SE statement employing the example of an unmarried bachelor given above).^19^ To illustrate, the following context was provided for the SE statement “This is a lake, but by that I'm not saying that it consists of water”:

Please imagine that Sally is exploring an area with high volcanic activity with Janet when they come across a pit filled with lava.

The following context was provided for the EC statement “Steve is responsible for the window breaking, but by that I am not saying that Steve caused the window to break”:

Please imagine that Steve and Harry play football in the garden. Steve and Harry get into a fight, and Steve pushes Harry such that Harry falls into a glass window and the window breaks.

And for the REC statement “John caused the file to be deleted, but by that I am not saying that John is responsible for the file being deleted”:

Please imagine that Chris works at a company with Bob. The company has instituted a new policy against deleting company files, but not everyone received the email informing them of the new policy, including another coworker, John. The next morning, John deletes a file.

For all contexts and statements, participants were subsequently presented with the question, “Does [agent] contradict herself?” They were then required to indicate their response using a 9-point Likert scale anchored at 1 with “definitely not” and at 9 with “definitely yes”.

If the objection is correct, then we would expect that the exculpatory contexts provided for the REC statements would illustrate how the agent could descriptively bring something about without being normatively responsible for that outcome, leading participants to treat the statements as non-contradictory. These contexts should not alter responses to the EC statements, however, since on the standard view responsibility entails causation.

The picture is somewhat more complicated with regard to the responsibility account. The reason is that, as noted above, this account focuses on the *dominant* ordinary use of causal attributions and responsibility attributions; it does not rule out, however, that there are other uses or that participants could be induced to read them in other ways for pragmatic reasons. As such, the account allows that the right context might lead people to infer that one of the attributions in our statements was intended in an alternative sense. Nonetheless, we would expect that this could occur for either the REC or the EC statements, and indeed that it could occur for the SE statements as well.

**Fig. 4:**
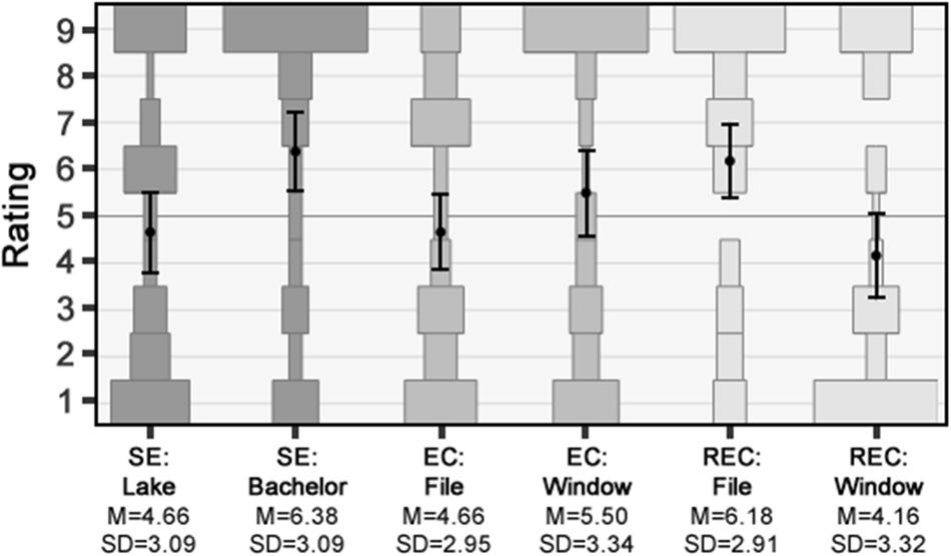
Results of Study 3. Plots show the relative percentage of participants selecting each response option, with means and 95% confidence intervals overlaid

The same recruitment method and pre-selection criteria were used as in our main studies. Results were collected from 100 participants (51.0% women, average age 39*.*3 years) and are shown in Fig. 4. Unlike the previous studies, with the added context we now see notable variation within each type of statement, with the mean for one statement in each pair being above the midpoint while the other is below. Indeed, the mean responses are significantly different between the two SE statements [*t*(98) = 2.78, *p* = 0.0064, *d* = 0.56] and the two REC statements [*t*(96.34) = 3.23, *p* = 0.0017, *d* = 0.65]. This is in line with the concern noted above that the context provided could shift how people read key terms in the statements. Importantly, this includes not just the causal attributions and the responsibility attributions, but also the Semantic Entailment statement involving “lake”: While participants in our previous studies tended to treat this statement as being contradictory, with the added context participants were now more likely to treat it as non-contradictory, although the mean response was not significantly below the midpoint [*M* = 4.66, *SD* = 3.09; *t*(49) = −0.78, *p* = 0.22 (one-tailed), *d* = 0.11]. This is consistent with switching from a dominant sense of “lake” that stipulates that it is an area of water (e.g., “a body of fresh or salt water of considerable size, surrounded by land”) and a secondary sense that allows for other types of liquid (e.g., “any similar body or pool of other liquid, as oil”).^20^

Overall, the results suggest against the objection. Against the critic’s expectations, participants continued to treat the REC statement concerning the deleted file as being contradictory despite the exculpatory context provided, with the mean response remaining significantly above the midpoint [*M* = 6.18, *SD* = 2.91; *t*(49) = 2.87, *p* = 0.0031 (one-tailed), *d* = 0.41]; further, participants no longer tended to treat the EC statement concerning the broken window as being contradictory, with the mean response now being below and not significantly different from the midpoint [*M* = 4.66, *SD* = 2.95; *t*(49) = −0.82, *p* = 0.42 (two-tailed), *d* = 0.12]. In response, the critic might note that the prediction held for the other REC statement, for which the mean response was now significantly below the midpoint [*M* = 4.16, *SD* = 3.32; *t*(49) = −1.79, *p* = 0.040 (one-tailed), *d* = 0.25], while the mean response for the other EC statement remained above the midpoint, if not significantly different from it [*M* = 5.50, *SD* = 3.34; *t*(49) = 1.06, *p* = 0.29 (two-tailed), *d* = 0.15]. Given this variation between statements, further testing is called for. That said, providing exculpatory context did *not* differentially lower ratings for the REC statements compared to the EC statements as the objection predicts. Indeed, a one-way ANOVA with type of statement as a within-participants factor showed no significant difference between the REC, EC, and SE statements [*F*(2,294) = 1.14, *p* = 0.32, η^2^ = 0.008], suggesting that what impact there was for context might be due to a more general shifting of how participants interpreted the key terms in the statements.

### Objection 2: other relationships

The second worry we consider is that our methodology is unfit to provide *conclusive* evidence for an “entailment relation” because it doesn’t rule out other potential relationships between causation and responsibility. And this is a reasonable point. The high contradiction ratings observed indicate that responsibility is not merely conversationally implicated by causation, but there remain alternative possibilities besides semantic entailment: Causation could merely presuppose responsibility or could conventionally implicate responsibility. While these are possible explanations of our findings, we believe that they are far less likely.

The first thing to note is that not only were the contradiction ratings for the REC statements very high across our main studies, but they were not statistically significantly different from those for the EC statements. Indeed, the distributions of responses for EC and REC are extremely similar. The most parsimonious explanation of such a pattern is that whatever relationship holds between responsibility and causation also holds between causation and responsibility (see Sytsma, 2021 for a related argument). A quite plausible candidate for this relationship is mutual entailment. By contrast, that causation and responsibility mutually presuppose one another seems far less plausible. In fact, it is difficult to conceptualize what such *mutual* presupposition would even mean.

Perhaps a more viable alternative is to argue that responsibility and causation stand in a relationship of mutual conventional implicature to one another. The basic idea, here, is that the relation is something that is inferred, but not based on features of the conversational context; rather it is something that is inferred from the meaning of the sentence used. For instance, from “the queen is English and therefore brave” we infer that being brave follows from being English (Davis, 2019, Sect. 2, based on Grice, 1989, p. 25), even though being brave would not seem to be part of the meaning of “English”. It is not clear how straightforwardly conventional implicature applies to our EC and REC statements. The most tempting option is perhaps to argue that our results reflect the meaning of the causal attributions (e.g., “John caused the file to be deleted”) rather than telling us about the ordinary concept of causation. The difficulty here is that both the standard view and the responsibility account are focused on causal attributions, as noted in Sect. 1. As such, this objection would seem to at best result in a pyrrhic victory, with our findings still providing evidence against the standard view and for the responsibility account.

At this point, a critic might be unsatisfied with our “inference to the best explanation”, arguing that despite the similarity in ratings for the EC and REC statements, these reflect different relations. Indeed, the fact that response patterns are statistically indistinguishable does not establish that we are dealing with the same relation. A presupposition relation and a semantic entailment relation, for instance, *might* happen to generate the same pattern of responses. But let us consider this example. An advocate of the standard view would need to argue that while responsibility entails causation, causation merely presupposes responsibility. This does not seem like a viable option, however, since a presupposition of a concept is necessarily poorer in information than the concept presupposing it and, thus, cannot at the same time entail the more information-rich statement. Nor would this seem to work any better if the critic were to instead claim that responsibility attributions conventionally implicate causal attributions. Finally, for the reasons discussed above, it does not seem that the critic would fare any better by explaining one pattern of results in terms of semantic entailment and the other in terms of conventional implicature, given the focus on causal attributions for specifying the concept of causation at issue for the Entailment Claim.

## Footnotes

1 Note that we often form negative judgments of people for merely *trying* to do something, even if they don’t succeed in bringing about the intended outcome. And such judgments might include blame. If so, it could then be argued that in such cases we blame a person in the absence of any causal involvement, as their action didn’t cause the intended outcome. We set this aside here, however, as the Entailment Claim we’re after is only concerned with responsibility for *outcomes*.

2 Taking omissions to be causally impotent, it has sometimes been suggested that omissions constitute a counterexample to the Entailment Claim. While some metaphysical theories of causation exclude the possibility of causation by omissions, those adopting such a view usually conclude that we cannot be blamed for the outcomes of our omissions. Others, such as Driver, explicitly include omissions in the Entailment Claim: “if an agent A is morally responsible for an event e, then A performed an action or omission that caused e” (2007, p. 423, for other accounts that consider EC and omissions compatible, see, e.g., Henne et al., 2017; McGrath, 2005; Willemsen, 2018).

3 It should be noted that not all philosophical work on causation targets the ordinary concept, although much does. Further, even for work that aims to develop a technical concept, the ordinary concept often remains relevant (Hall & Paul, 2003).

4 Most experimental work on the impact of norms on causal judgments has followed the theoretical literature and investigated causal attributions. However, researchers have also used other constructions, such as statements employing “made” (Samland et al., 2016) or “because” (Kominsky et al., 2015; Livengood and Machery, 2007; Livengood et al., 2017). Other work has investigated causatives like “break” and “burn” (Rose et al., 2021; Schwenkler & Sievers, 2022) and potential synonyms of “cause” (Sytsma et al., 2019). The results so far seem to be mixed in that some authors highlight interesting differences, while others suggest that the differences are minor.

5 Psychological models share these latter two assumptions, taking judgments about causation to precede those of moral responsibility (e.g., Fincham & Shultz, 1981; Heider, 1958; Schleifer et al., 1983; Shaver, 1985).

6 See, for example, Alicke (1992); Knobe & Fraser (2008); Hitchcock & Knobe (2009); Sytsma et al. (2012); Reuter et al. (2014); Kominsky et al. (2015); Henne et al. (2017); Willemsen (2018); Willemsen & Reuter (2016); Livengood et al. (2017); Kominsky & Phillips (2019); and, Livengood & Sytsma (2020), among many others. For an overview, see Willemsen & Kirfel (2019).

7 This includes explanations that focus on how people think about counterfactuals (e.g., Halpern & Hitchcock, 2015; Hitchcock & Knobe, 2009; Icard et al., 2017; Kominsky & Phillips, 2019; Kominsky et al., 2015), explanations that contend the desire to blame or praise biases people’s causal judgments (e.g., Alicke, 1992, 2000; Alicke et al., 2011; Rose, 2017), and explanations that hold that the experimental results are due to pragmatic factors (e.g., Samland & Waldmann, 2016; Samland et al., 2016).

8 See Sytsma (2022a) for a recent survey of the evidence for this view relative to other accounts.

9 See Sarin et al. (2017), Murray and Lombrozo (2017), Grinfeld et al. (2020), Sytsma & Livengood (2021), and Sytsma (2021, 2022b, forthcoming).

10 Preregistration at https://osf.io/hx735; online repository at https://osf.io/kv9rt.

11 The mean rating for the contradictory statement were significantly above the midpoint [M = 8.31, SD = 1.86, *t*(70) = 14.96, *p* < .001 (one-tailed), *d* = 1.78], while the mean rating for the non-contradictory statement was significantly below the mid-point [M = 1.99, SD = 1.92, *t*(70) = -13.03, *p* < .001 (one-tailed), *d* = 1.57].

12 One-way ANOVAs did not show a significant effect for the different causal attributions for either REC [*F*(2,68) = 1.24, *p* = .30, η^2^ = .035] or EC [*F*(2,68) = .33, *p* = .72, η^2^ = .01].

13 Note, that discussions about the entailment claim often use “being a cause of X”, “having caused X”, and “being causally responsible for X” interchangeably. Sartorio, for instance, defines the entailment claim in the following way: “An agent A is morally responsible for an outcome O only if A is *causally responsible* for O, i.e., only if one of A’s actions or omissions *caused* O; moreover, the fact that one of A’s actions or omissions *caused* O (partly) explains the fact that A is morally responsible for O” (Sartorio, 2007, p. 750, own emphasis).

14 https://osf.io/3rgxs

15 Contradictory [M = 8.47, SD = 1.68, *t*(71) = 17.56, *p* < .001 (one-tailed), *d* = 2.07], Noncontradictory [M = 2.28, SD = 2.33, *t*(71) = -9.93, *p* < .001 (one-tailed), *d* = 1.17].

16 As there were once again no significant effects of the different outcomes for either REC [*F*(2,69) = .035, *p* = .97, η^2^ = .001] or EC [*F*(2,69) = 2.28, *p* = .11, η^2^ = .062], we collapsed the data for the different outcomes in the subsequent analysis.

17 https://osf.io/f3zud

18 Contradictory [*t*(70) = 10.80, *p* < .001 (one-tailed), *d* = 1.28]; Non-contradictory [*t*(70) = 4.40, *p* < .001 (one-tailed), *d* = .52]; Semantic Entailment [*t*(70) = 4.98, *p* < .001 (one-tailed), *d* = .59]; Conversational Implicature [*t*(70) = -9.37, *p* < .001 (one-tailed), *d* = 1.11]; Control [*t*(70) = -9.14, *p* < .001 (one-tailed), *d* = 1.08].

19 Full materials can be accessed through the online repository: https://osf.io/kv9rt

20 Definitions taken from https://dictionary.com/browse/lake, accessed 5 February 2023.
